# Antibody-conjugated gold nanoparticles as nanotransducers for second near-infrared photo-stimulation of neurons in rats

**DOI:** 10.1186/s40580-022-00304-y

**Published:** 2022-03-21

**Authors:** Jiansheng Liu, Jiajia Li, Shu Zhang, Mengbin Ding, Ningyue Yu, Jingchao Li, Xiuhui Wang, Zhaohui Li

**Affiliations:** 1 Department of Neurology, Zhuhai People’s Hospital, Zhuhai Hospital of Jinan University, Zhuhai, Guangdong 519000 People’s Republic of China; 2 Shanghai Engineering Research Center of Nano-Biomaterials and Regenerative Medicine, College of Chemistry, Chemical Engineering and Biotechnology, Donghua University, Shanghai, 201620 People’s Republic of China; 3 Department of Neurology, Shanghai Eighth People’s Hospital, Shanghai, 200233 People’s Republic of China; 4 Institute of Translational Medicine, Shanghai University, Shanghai, 200011 People’s Republic of China

**Keywords:** Gold nanoparticles, Polydopamine, Near-infrared, Neural stimulation, Photothermal transducers

## Abstract

Infrared neural stimulation with the assistance of photothermal transducers holds great promise as a mini-invasive neural modulation modality. Optical nanoparticles with the absorption in the near-infrared (NIR) window have emerged as excellent photothermal transducers due to their good biocompatibility, surface modifiability, and tunable optical absorption. However, poor activation efficiency and limited stimulation depth are main predicaments encountered in the neural stimulation mediated by these nanoparticles. In this study, we prepared a targeted polydopamine (PDA)-coated gold (Au) nanoparticles with specific binding to thermo-sensitive ion channel as nanotransducers for second near-infrared (NIR-II) photo-stimulation of neurons in rats. The targeted Au nanoparticles were constructed via conjugation of anti-TRPV1 antibody with PEGylated PDA-coated Au nanoparticles and thus exhibited potent photothermal performance property in the second NIR (NIR-II) window and converted NIR-II light to heat to rapidly activate Ca^2+^ influx of neurons in vitro. Furthermore, wireless photothermal stimulation of neurons in living rat successfully evoke excitation in neurons in the targeted brain region as deep as 5 mm beneath cortex. This study thus demonstrates a remote-controlled strategy for neuromodulation using photothermal nanotransducers.

**Supplementary Information:**

The online version contains supplementary material available at 10.1186/s40580-022-00304-y.

## Introduction

Dysfunction of neural activity is closely related with neurological and psychiatric disorders such as epilepsy [1], Parkinson’s disease (PD) [2], and depression [3]. Regulation of neural activity not only serves as cornerstone for exploring the function and interactions of neural network in basic neuroscience, but also emerges as an evolving treatment modality for various neurological disease in neurology [4].

Small molecules that target ion channels (e.g., Na^+^, K^+^, and Ca^2+^) and neurotransmitters can be used to pharmacologically modulate the neuronal activity [5]. Some of them have successfully entered into clinic from laboratory bench. Nevertheless, the pharmacokinetic and metabolic profiles of these drugs are difficult to control, leading to unavoidable off-target side effects after systematic administration. In contrast, modulation of neural activity by physical stimulus (e.g., electric current, magnetic field, and ultrasound) offers the unique advantages of strong accessibility and controllability. For instance, with the assistance of magnetic nanoparticles (MNPs), alternating magnetic field could excite action potentials in transient receptor potential vanilloid 1 (TRPV1)-expressed neurons in the targeted brain regions and therefore evoke specific motor behaviors in awake mice [6–8]. In another study, magnetic activation of TRPV4-overexpressed neurons can also be achieved by anti-His antibody modified MNPs [9]. Ultrasound has also been applied to modulate neural activity directly or indirectly through piezoelectric materials [10–13]. However, there are still major shortcomings in these techniques including the inflammation and gliosis due to the implantation of stimulation electrodes [14] and low spatiotemporal resolution of transcranial stimulation [13, 15]. Thus, development of alternative approaches toward mini-invasive yet precise modulation of neural activity remains great challenge.

Light is another physical stimulus modality that has been widely used for bioregulation due to its biosafety and remote spatiotemporal controllability [16–18]. Compared with the above techniques, the optical stimulation holds the distinguished advantage of high spatiotemporal and neurochemical resolution. Optogenetics, which exploit microbial opsins such as channelrhodopsin (ChR), halorhodopsin (NpHR), and archaerhodopsin (Arch), provide a powerful tool for precise control of neurons and even specific behaviors in living rodents. However, opsins and their derivatives only respond to the visible light ranging from ~ 470 to ~ 630 nm [19]. Due to the shallow tissue penetration depth of the light in visible spectrum, most optogenetic stimulation methods still need implantation of optical fibers, which also result in inflammation and gliosis at the implant site [10]. The discovery of biologically transparent near-infrared (NIR) light source (650–1350 nm) with less photon scattering and deeper tissue penetration offers opportunities for photoregulation in deep tissue [18, 20]. Upconversion nanoparticles (UCNP) consisting of a host crystal and lanthanide dopants (e.g., Yb^3+^/Tm^3+^, Yb^3+^/Er^3+^, Yb^3+^/Ho^3+^) could absorb NIR light and convert it into visible light. Thus, UCNP-mediated optogenetics for wireless stimulation of cultured neural cells has been extensively investigated [21–23]. However, this kind of optogenetics still require manipulation of targeted cells via genetic transfection. The discovery of neuronal thermosensitive channel and the advent of photothermal nanotransducers open the possibility of wireless neural regulation without genetic modification [24]. TRPV1 is one of the best characterized thermosensitive channels first cloned from rat dorsal root ganglia [25]. As a non-selective cation channel with high Ca^2+^ permeability, it has a wide distribution in the peripheral and central nervous system [26]. Except for its well-established role in nociception and heat sensing, TRPV1 is now recognized to have a broader function and its potential as a drug target for the treatment of several neurological disorders (e.g., chronic pain, addiction, anxiety) is under intensive investigation [27]. Optical nanomaterials acting as photothermal nanotransducers can convert NIR light into local mild heat, allowing temperature-sensitive ion channels (e.g., TRPV1) on neuronal membrane to be activated [28]. Gold nanomaterials are one of the most reportedly used photothermal transducers in regulating neural activity [29–32]. Carbon nanohorns [33] and semiconducting polymer nanoparticles [34] have also been reported to be able to modulate the neural activity through photothermal effects induced by NIR. Despite these achievements, the NIR light used in these studies was in the first NIR (NIR-I) window (650–950 nm) that still shows limited tissue penetration. Therefore, NIR-I mediated neural modulation are mostly confined to an in vitro investigation tool. In recent years, it has been well established that light in the second NIR (NIR-II) window (1000–1350 nm) has a stronger penetrating capability and higher power safety limit compared to NIR-I [35–37]. However, no direct evidence is yet available for developing a strategy to optimize NIR-II light-mediated neuromodulation.

**Fig. 1:**
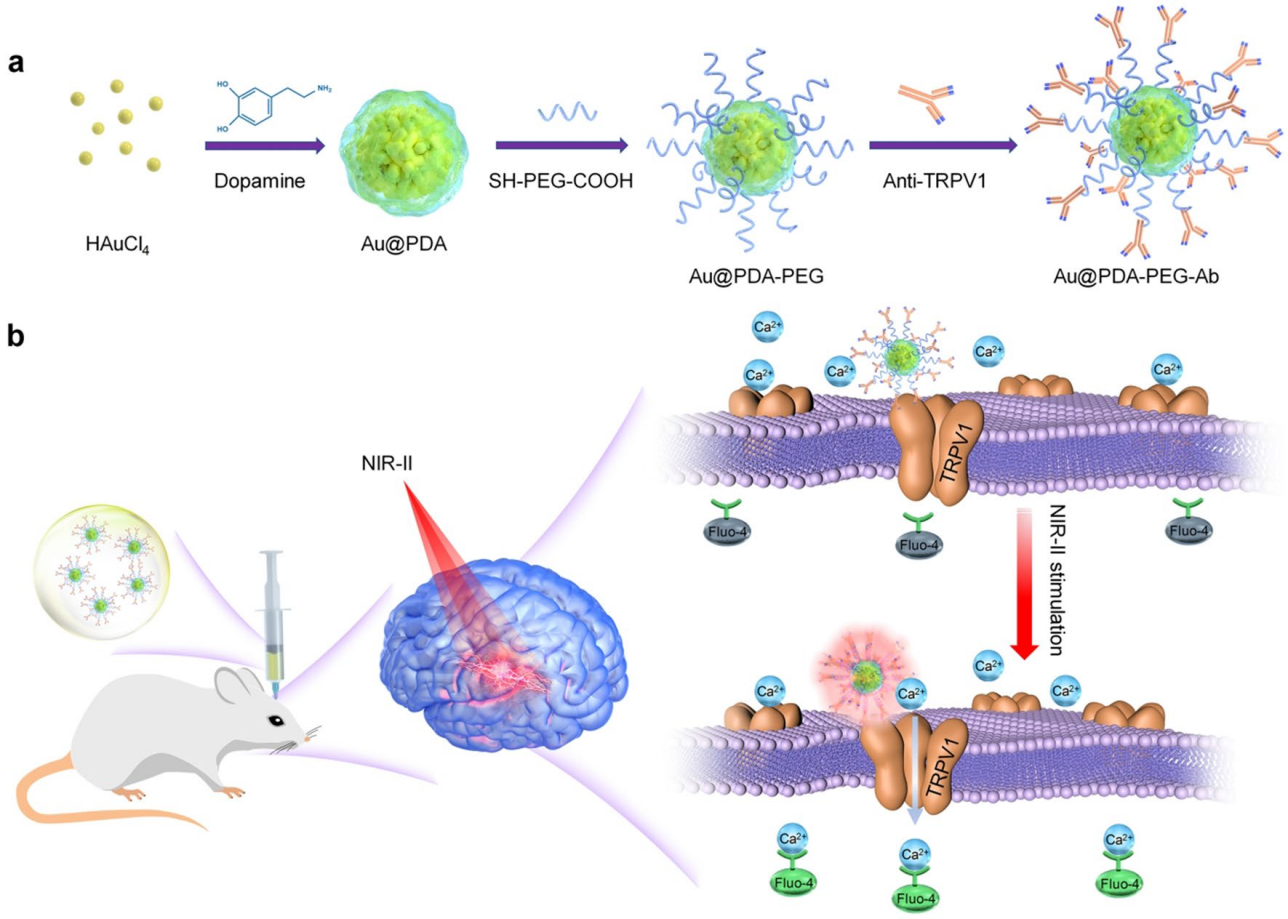
Scheme of NIR-II neural stimulation via targeted Au nanoparticles. **a** Preparation of antibody-conjugated gold nanoparticles. **b** Mechanisms of nanoparticle-mediated NIR-II neural stimulation

In this context, we reported a targeted Au nanoparticle to photothermally modulate neuronal activity via NIR-II irradiation. The Au nanoparticles were facilely prepared by mussel-inspired polydopamine coating. Self-polymerized polydopamine by dopamine could be successfully coated onto the Au nanoparticles, which not only imparts them with robust and tunable surface chemistry property, but also helps to enhance thermal stability [37, 38]. The anti-TRPV1 antibody was used to further modify the surface of the nanoparticles (Fig. 1a). The as-prepared nanoparticles displayed excellent biocompatibility, targetability and photothermal conversion property in the NIR-II window. Then, we tested the ability of Au nanoparticle-mediated infrared neural stimulation by NIR-II laser irradiation. Our results showed that TRPV1 channels on cultured neurons can be successfully stimulated. Notably, this study also demonstrated that this wireless strategy could remotely activate the neurons located 5 mm beneath the cortex in living rats (Fig. 1b). Hence, Au nanoparticles fabricated in the present study exhibited promising potential as a photothermal nanotransducer for NIR-II neural stimulation. Besides, the rationale for this work could be extrapolated to other NIR-II-absorbing photothermal agents by adjusting their parameters. Furthermore, this NIR-II neural stimulation system could be improved through rational design of brain targeting systems to deliver photothermal nanotransducers [39, 40]. This study thus offers an innovative approach to prompt the evolution of neuromodulation.

## Materials and methods

### Materials and reagents

Chloroauric acid (HAuCl_4_·3H_2_O, 48–50% Au) was purchased from Macklin Biochemical Co., Ltd (Shanghai, China). Carboxyl-poly(ethylene glycol)-thiol (COOH-PEG-SH, *M*w = 5 kDa) were purchased from Laysan Bio Inc (Arab, Alabama). Dopamine hydrochloride, tris(hydroxymethyl)aminomethane (TRIS), and bicine were purchased from Aladdin Bio-chem Technology Co., Ltd (Shanghai, China). High glucose Dulbecco’s modified Eagle medium (DMEM), fetal bovine serum (FBS), penicillin/streptomycin antibiotics, and Trypsin–EDTA (0.05%) were purchased from Gibco (Grand Island, NY, USA). N-hydroxy succinimide (NHS), 1-ethyl-3-(3-dimethyl-aminopropyl) carbodiimide hydrochloride (EDC·HCl) and bovine serum albumin (BSA) were obtained from Sigma-Aldrich (St. Louis, USA). Fluo-4 AM, cell counting kit-8 (CCK-8) and 4,6-diamidino-2-phenylindole (DAPI) were from Dalian Meilun Biotech Co. Ltd (Dalian, China). TRPV1 polyclonal antibody (92 kDa) was purchased from Bioss Inc (Boston, MA, USA). C-fos antibody, fluorescent probe-conjugated secondary antibodies, fluorescein isothiocyanate isomer I (FITC), terminal deoxyribonucleotidyl transferse (TdT)-mediated biotin-16-dUTP nick-end labelling (TUNEL) kit, and bicinchoninic acid (BCA) protein assay kit were purchased from Beyotime Biotechnology Co., Ltd (Shanghai, China). NIR-II laser (λ = 1064 nm) were purchased from Changchun Laser Optoelectronics Technology Co. Ltd (Jilin, China). Distilled water was obtained from Pall-cascada laboratory water system (PALL, USA). Other agents were purchased from Sinopharm Chemical Reagent Co., Ltd (Shanghai, China) unless otherwise stated.

### Cell culture and animals

The immortalized mouse hippocampal cell line (HT-22) and human neuroblastoma cells (SH-SY5Y) were obtained from Cell Bank, Chinese Academy of Sciences (Shanghai, China). Sprague–Dawley (SD) rats (male, 220–300 g) were purchased from Shanghai Jie Si Jie Laboratory Animal Co., Ltd (Shanghai, China). All animal experiments were performed according to the protocols approved by the Institutional Animal Care and Use Committee (IACUC) at Donghua University.

### Synthesis of polydopamine coated gold nanoparticles

The polydopamine (PDA) coated gold nanoparticles (Au@PDA) were prepared by a one-pot method [37]. In brief, dopamine hydrochloride at a concentration of 4 mg/mL with variable volumes were added into 72 mL TRIS buffer (10 mM, PH = 8.5) under vigorous stirring. After stirring for 3 min, 7.2 mL of HAuCl_4_ (1 mg/mL) was injected into the solution. The reaction solution was further vortexed for 8 h. The as-prepared Au@PDA nanoparticles were centrifugated (15,000 rpm, 15 min) and purified with ultrapure water three times. The obtained nanoparticles were dissolved in water and stored at 4 °C for further use.

To improve the stability of PDA coated gold nanoparticles, polyethylene glycol (PEG) was used to modify the surface of nanoparticles. PDA coated gold nanoparticles (1 mg) were dispersed in 10 mL of bicine buffer (10 mM, pH = 8.5) with stirring, followed by adding 10 mg of COOH-PEG-SH. The reaction was continued for 36 h at room temperature in the dark. The Au@PDA-PEG were obtained after centrifugation and washing with distilled water thrice. The purified products were stored in H_2_O at 4 °C for further use.

To bestow nanoparticles with specific cell targetability, Au@PDA-PEG was further modified with anti-TRPV1 antibody, 500 µg of the carboxylated Au nanoparticles were centrifugated and re-suspended in MES solution (pH = 5.0). Then, 10 equiv of NHS and EDC•HCl were added to activate the carboxylic groups of the PEGylated gold nanoparticles by reacting for 30 min with gentle stirring. The excess EDC and NHS were removed via centrifugation (15,000 rpm, 5 min). Then, the activated PEGylated gold nanoparticles were re-suspended in 2 mL PBS buffer (pH 7.4) and 10 µg of antibody was added into the mixture with stirring overnight at 4 °C. The obtained nanoparticles (Au@PDA-PEG-Ab) were purified by centrifugation and stored in PBS for further use.

For the fluorescent probe labelled nanoparticles, FITC was covalently coupled to the amines of TRPV1 antibody. Antibody and FITC were mixed at a protein to FITC molar ratio of 1:5 in sodium carbonate buffer (0.1 M, pH = 9.0) and stirred overnight at 4 °C. The reactant was dialyzed against distilled deionized water three times to remove unreacted FITC (molecular cutoff = 3000). Then, the FITC-labelled Au@PDA-PEG-Ab nanoparticles were synthesized using the same procedure for the preparation of Au@PDA-PEG-Ab. All the experiments were performed in the dark.

### Characterization of nanoparticles

Dynamic light scattering (DLS) and zeta potential were performed by Zetasizer Nano ZS analyzer (Malvern, UK). The morphology of nanoparticles was observed with a transmission electron microscope (TEM, Talos F200S, FEI, USA). UV–vis spectrum was performed by a TU-1810 UV–vis spectrophotometer (Persee, Beijing, China). Fluorescence spectra were recorded on RF-6000 fluorescence spectrophotometer (Shimadzu, Japan). The Au contents in nanoparticles were measured by Leeman Prodigy Series inductively coupled plasma atomic emission spectrometry (ICP-AES, USA).

### In vitro photothermal performance of Au nanoparticles

To obtain the photothermal conversion efficiency of Au nanoparticles upon NIR-II irradiation, 2 mL nanoparticle disperse solutions at a concentration of 50 µg/mL were irradiated by NIR-II laser (1.5 W/cm^2^) for 30 min and cooled naturally to room temperature. The photothermal conversion efficiency of the as-prepared Au nanoparticles was calculated according to previous method [34]. To evaluate the photothermal performance of Au nanoparticles with different concentrations, 100 µL of Au@PDA-PEG-Ab solutions at different Au concentrations (10, 25, 50, and 100 µg/mL) were illuminated by NIR-II laser (1064 nm, 1.0 W/cm^2^) for 4 min. The temperature change at 30 s intervals was monitored through infrared thermal imaging camera (FOTRIC, Shanghai, China). NIR-II laser with different power intensities (0.5, 1.0, 1.5 W/cm^2^) at fixed Au concentration (50 µg/mL) was also evaluated. Besides, NIR-II photothermal performance of Au@PDA-PEG-Ab was compared with that of Au@PDA and Au@PDA-PEG. The photothermal stability of Au nanoparticles was evaluated by performing 5 cycles of on/off NIR-II laser irradiations. The Au nanoparticles (50 µg/mL) were illuminated with NIR-II laser (1.0 W/cm^2^) for 4 min and then cooled to room temperature naturally in each cycle.

### In vitro cytotoxicity assay

CCK-8 assay was applied to evaluate the biocompatibility of as-prepared Au nanoparticles on HT-22 cells. Cells were seeded at a density of 1 × 10^4^/well on 96-well plates and incubated in culture medium for 24 h. Afterwards, cells were treated with Au@PDA-PEG-Ab with variable concentrations (25, 50, 100, and 200 µg/mL) for 24 h. Then, CCK-8 solution was added into the DMEM medium (1:10, v/v) to evaluate the cell viability.

The effects of NIR irradiation on cell viability were also tested. After 24 h of attachment, cells were treated with Au@PDA-PEG-Ab (50 µg/mL) for 12 h and then irradiated by 1064 nm laser (0.5 W/cm^2^ or 1.0 W/cm^2^) for different time intervals (5, 30, and 60 s). After re-incubation for another 12 h, cell viability was evaluated by CCK-8 assay.

### Cellular uptake study

HT-22 or SH-SY5 cells were seeded into 12-well cell plates and allowed to adhere for 24 h in a humidified atmosphere (95% O_2_/5% CO_2_) at 37 °C. The cells were treated with FITC labelled Au@PDA-PEG-Ab at a concentration: 50 µg/mL) for 4 h in culture medium. Then the cells were fixed by 4% paraformaldehyde and stained by DAPI. The images of the cells were obtained using a fluorescence microscope (Leica DMi8, Germany).

### Detection of TRPV1 in vitro and in vivo

TRPV1 expression of cells was evaluated by western blot assay. HT-22 or SH-SY5 cells were incubated in T25 flasks for 24 h. Then, the cells were lysed and collected after centrifugation. After determining the total protein via BCA protein assay reagent, the proteins of each sample were separated via sodium dodecyl sulfate polyacrylamide gel electrophoresis (SDS-PAGE) and then electrically transferred to polyvinylidene fluoride (PVDF) membranes. The membranes were blocked with 5% non-fat milk in tris-buffered saline containing 0.5% TBST (tris-buffered saline with Tween) for 1 h and incubated overnight with primary antibodies (TRPV1 and GADPH, 1:800 and 1:1000 dilutions, respectively) at 4 °C. The membranes were washed three times with TBST and then incubated with secondary antibody for 30 min at room temperature. The membranes were stained by an electrochemiluminescence detection kit and imaged using a gel imaging system (Tanon, Biotech, Shanghai, China).

To detect TRPV1 expression in murine brain, rat was euthanized to extract brain. After fixing in 4% paraformaldehyde, rat brain was dehydrated with gradient sucrose solution (15% and 30%) for 24–48 h, then embedded in frozen optimal cutting temperature (O.C.T.) medium and sectioned into slices at a thickness of 10 μm (Leica cryostat, Germany). Sections were re-hydrated with PBS containing 0.3% Triton X-100 (PBST), rinsed with 10% BSA solution for permeabilization, and stained with anti-TRPV1 antibody solution (1:300) at 4 °C overnight. Then the sections were washed with PBS and stained with cy3-conjugated rabbit secondary antibody at room temperature for 50 min protected from light. DAPI was used for nuclei staining. The stained sections were observed under LSM710 confocal laser scanning microscope (Carl Zeiss, Germany).

### Photothermal stimulation of hippocampal neurons

HT-22 cells were incubated with Au@PDA-PEG-Ab (50 µg/mL) in 12-well plates for 24 h. Then cells were washed with PBS three times and stained with 5 µM of Fluo-4 AM diluted in HBSS for 20 min. fivefold volume of HBSS containing 1% FBS was added to incubate for another 40 min. Afterwards, calcium imaging buffer (10 mM HEPES, 137 mM NaCl, 5 mM KCl, 1 mM CaCl_2_, 0.5 mM MgCl_2_, 5 mM glucose, 0.1% BSA, pH adjusted to 7.4) was used to wash and incubate the cells. A 1064-nm NIR laser beam from a continuous wave diode laser (0.5 W/cm^2^) was incorporated into the inverted fluorescence microscopy system (Nikon Eclipse Ti-S, Japan). Laser beam was focused on the target cells using 20× objective lens. The green fluorescence of Fluo-4 AM was monitored and recorded in real-time using blue-light excitation. ImageJ software (Fiji) was used to analyze the fluorescence intensities.

Photothermal stimulation of hippocampal neurons in vivo was also evaluated. After anesthesia, 4 µL of PBS or Au nanoparticles (100 µg/mL) was stereotactically injected into the hippocampus of rat (anterior–posterior [AP], − 5.0 mm; medial–lateral [ML], ± 5.0 mm; dorsal–ventral [DV], − 5.0 mm). 10 s pulses of alternating NIR-II irradiation at 1.0 W/cm^2^ were delivered through burr holes with 50 s rest intervals in between over the course of 10 min. Rats were sacrificed after 90 min to allow for immunofluorescent detection of c-fos expression [41]. Hematoxylin and eosin (H&E) and TUNEL staining were also performed to evaluate the potential side lesions induced by NIR irradiation.

### Electrophysiological recording

Acute brain slices were prepared according to our previously published method [42]. Briefly, brain was rapidly extracted and immersed in ice-cold artificial cerebrospinal fluid (ACSF) containing 213 mM sucrose, 2.5 mM KCl, 26 mM NaHCO_3_, 1.25 mM NaH_2_PO_4_, 2 mM CaCl_2_, 2 mM MgCl_2_, and 10 mM Glucose, bubbled with 95% O_2_/5% CO_2_. Coronal brain slices (~ 250 μm) were cut on a Vibratome 1000PLUS, and then incubated in oxygenated ACSF for ~ 30 min at 34 °C prior to recording. For electrophysiological recording, a slice was transferred to a recording chamber under constant perfusion of oxygenated ACSF with or without Au@PDA-PEG-Ab (100 µg/mL) at a flow rate of 1.5–2 mL/min. Whole-cell current clamp recordings were performed with glass pipettes (3–5 MΩ) containing the following solution (in mM): 135 K-gluconate, 4 KCl, 10 HEPES, 10 Na-phosphocreatine, 4 MgATP, 0.4 Na_2_GTP, and 1 EGTA (with pH 7.2–7.3, and osmolarity ~ 295 mOsm/L) [43]. Currents were injected into neurons with 10 pA increments from -100 pA to 140 pA in current clamp. NIR irradiation (1.0 W/cm^2^) was applied as needed. Recordings were made using 700B amplifier (Axon Instruments, Union City, CA); data were sampled at 10 kHz and filtered at 4 kHz and collected using pCLAMP software (Molecular Devices, Sunnyvale, CA, USA).

## Results and discussion

### Preparation and characterization of TRPV1 antibody-conjugated gold nanoparticles

As a reducing agent, dopamine catechol can be oxidized to quinone by chloroauric acid in a weak alkaline environment. Those quinones could further react with catechol or other quinones to form polydopamine (PDA), which coats Au as a capping agent [44, 45]. PDA-coated gold (Au) nanoparticles (Au@PDA) were prepared by a simple one-pot method as described previously [37]. HAuCl_4_ and dopamine were reacted in alkaline TRIS solution (pH = 8.5). The color of mixture turned from brown to black as the reaction proceeds (Additional file 1: Fig. S1). Consistent with previous findings [37], the different mass ratios between dopamine and HAuCl_4_ could affect the particle size and zeta potentials of the nanoparticles. In the case of fixed HAuCl_4_ content, increasing the concentration of dopamine can increase the UV absorption of the nanoparticles (Additional file 1: Fig. S2a), which may be due to the abundance of reductants. However, the nanoparticles formed at a mass ratio of 2:1 between dopamine and HAuCl_4_ (formula 3) had the smallest particle size and the most negative zeta potential after reacting for 8 h (Additional file 1: Fig. S2b-d). Therefore, Au@PDA was prepared in the ratio of formula 3 in the following study. The yield was measured to be 56.6% by measuring the amount of Au in the nanoparticle by ICP.

**Fig. 2:**
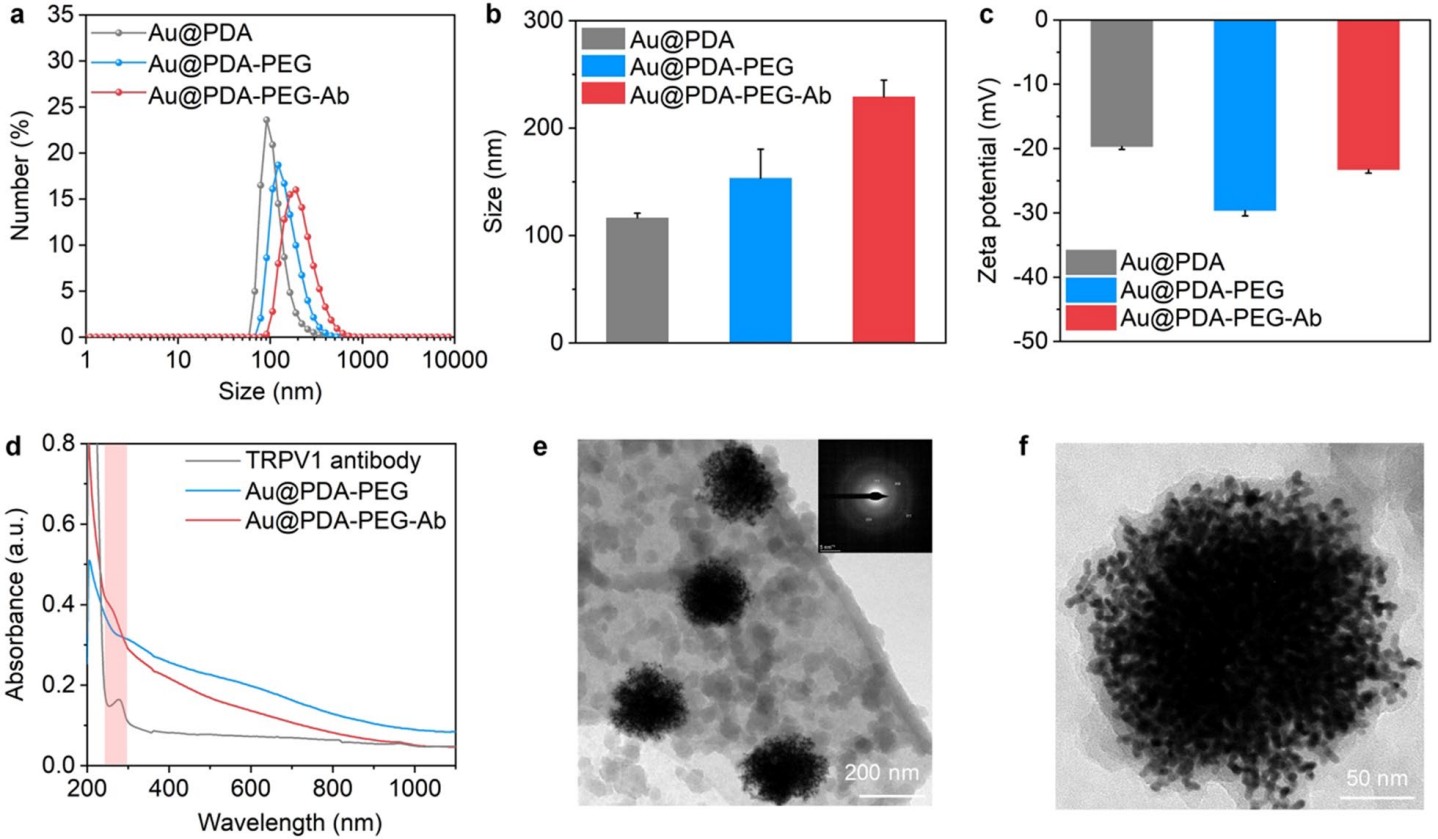
Characterization of Au nanoparticles. **a** Size distributions of Au@PDA, Au@PDA-PEG, and Au@PDA-PEG-Ab. **b** Hydrodynamic sizes of Au nanoparticles (n = 3). **c** Zeta potentials of Au nanoparticles (n = 3). **d** The UV–vis absorbance spectrum of TRPV1 antibody, Au@PDA-PEG, and Au@PDA-PEG-Ab. **e** TEM images of Au@PDA-PEG-Ab nanoparticles. Inset: SAED pattern of nanoparticles. **f** Enlarged view of Au@PDA-PEG-Ab nanoparticles

Previous studies have shown that gold nanoparticles prepared from borohydrate and sodium citrate can be polyethylene glycol-coated (PEGylated) through metal-S bonds on the surface of nanoparticles [46, 47]. As PDA deposits on Au surfaces, it offers more robust alternatives to bind with functional PEG via Michael addition and/or Schiff base reactions due to readily tailorable surface chemistry [44, 45, 48]. After functionalized with thiolated PEG (SH-PEG-COOH), the mean hydrodynamic size of Au nanoparticles increased from 116.4 to 153.3 nm (Fig. 2a, b). The zeta potential of PEGylated Au nanoparticles decreased from − 19.7 to − 29.6 mV (Fig. 2c), which could be ascribed to the presence of highly negatively charged carboxyl groups on the surfaces of particles.

To increase the targeting efficiency of Au-based nanoparticle, antibody was further modified on the nanoparticles. The carboxyl groups on the surface of nanoparticles endow the PEGylated PDA-coated Au nanoparticles with the capability to conjugate with amine-terminated antibodies. Anti-TRPV1 antibody was conjugated with the surface of nanoparticles using ethyl(dimethylaminopropyl) carbodiimide (EDC) and N-hydroxysuccinimide (NHS). The carboxyl groups on the surface of nanoparticles bind covalently to the amine groups of the antibody presumably through a carbodiimide coupling reaction. DLS showed that the resulting Au@PDA-PEG-Ab exhibited increased hydrodynamic diameter from 153.3 to 229.0 nm (Fig. 2a, b). The zeta potential of Au@PDA-PEG-Ab was − 23.3 mV, smaller than that of carboxylated Au@PDA-PEG (-29.6 mV) (Fig. 2c). Successful bioconjugation was also demonstrated by comparing UV–vis spectra of Au@PDA-PEG and anti-TRPV1 conjugated Au@PDA-PEG (Au@PDA-PEG-Ab), which reveal characteristic protein absorption at ~ 280 nm (Fig. 2d). The TEM images show that the as-synthesized nanoparticles display uniform spherical morphology with or without PEG and antibody modification (Fig. 2e and Additional file 1: Fig. S3). The selected area electron diffraction (SAED) pattern of nanoparticles demonstrates that Au nanoparticles with different surface modification exhibit similar crystalline structure (Fig. 2e and Additional file 1: Fig. S3), indicating the core–shell structure of as-prepared nanoparticles. Anisotropic deposition of Au atoms was induced due to the strong binding of polydopamine, which leads to consistent divergence of branches and eventual formation of disordered hyperbranched structures [37]. High-resolution TEM confirms that the nanoparticles were composed of hyperbranched short Au nanorods with PDA and PEG polymer coating on the surface of the nanoparticles (Fig. 2f). These results validated that anti-TRPV1 antibody can be successfully conjugated on the nanoparticle surface.

### Photothermal property of Au nanoparticles

**Fig. 3:**
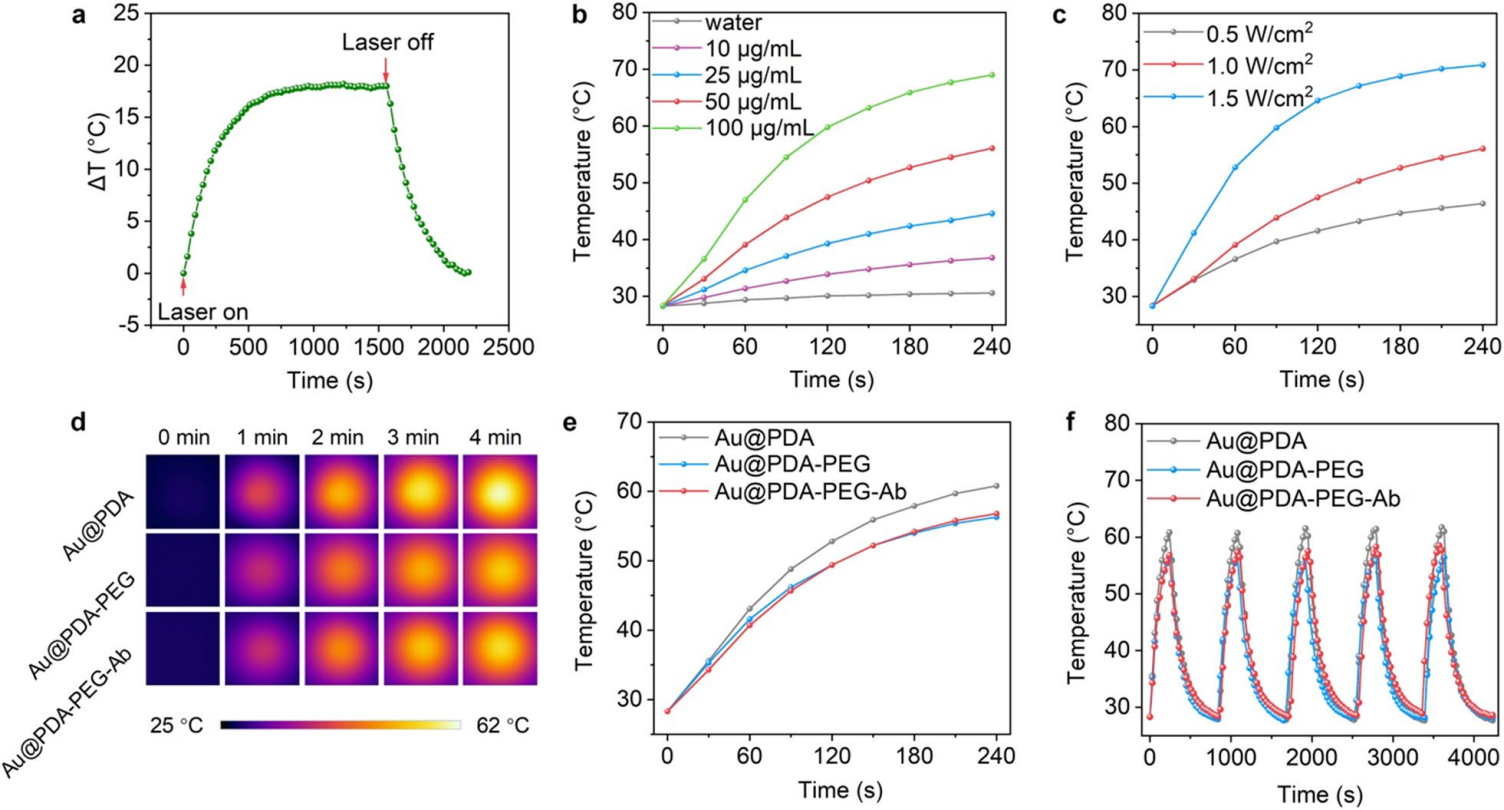
Photothermal performance of Au nanoparticles. **a** Temperature curve of Au@PDA-PEG-Ab solution (50 μg/mL, 2 mL) irradiated with a 1064-nm laser at a power density of 1.5 W/cm^2^. The laser was shut off after 30 min of irradiation. **b** Temperature curves of aqueous dispersion (100 μL) of Au@PDA-PEG-Ab at different concentrations from 10, 25, and 50 to 100 μg/mL under NIR-II laser irradiation (1.0 W/cm^2^). **c** Temperature curves of Au@PDA-PEG-Ab solution (50 μg/mL) under NIR-II laser irradiation at different power densities (0.5, 1.0, and 1.5 W/cm^2^). **d** Infrared thermal images of aqueous dispersion of Au@PDA, Au@PDA-PEG, and Au@PDA-PEG-Ab (50 μg/mL) upon NIR-II irradiation for 4 min. **e** Temperature curves of solutions in **d**. **f** Photothermal stability of Au@PDA, Au@PDA-PEG, and Au@PDA-PEG-Ab after 5 on/off cycles of laser irradiation (1.0 W/cm^2^). The concentration of nanoparticle dispersion was 50 μg/mL

The photothermal performance of as-prepared Au nanoparticles in NIR-II window was evaluated by measuring the temperature changes of Au nanoparticle suspensions upon NIR-II (λ = 1064 nm) laser illumination. The temperature of solution rises rapidly in the first 4 min of laser irradiation, and then slows down to enter a plateau after 10 min (Fig. 3a). Afterwards, the temperature of the solution could only be slightly increased by extending the illumination time. The temperature of the solution dropped in a specific manner after the light was turned off (Additional file 1: Fig. S5). Based on the previously described method [37], the calculated photothermal conversion efficiency is 80.0%. Then the effects of Au nanoparticle concentrations and laser power intensity on the photothermal performance of the nanoparticles were investigated. Under the same power, increasing the concentration of gold nanoparticles can enhance its heating effect. After 4 min of NIR-II irradiation, the temperature of Au nanoparticle solution at the concentration of 100 μg/mL can be raised to 69.0 °C, while gold nanoparticle solution at the concentration of 50 μg/mL can only be raised to 56.1 °C. However, when the concentration of gold nanoparticles was reduced to 10 μg/mL, the temperature rise of the solution is only 6.2 °C higher than that of water after NIR-II irradiation (Fig. 3b). When the concentration of nanoparticles was fixed at 50 μg/mL, changing the power of the laser can also significantly affect the heating effect of the solution. After 4 min of NIR-II irradiation with a power of 1.5 W/cm^2^, the solution temperature can reach 70.9 °C, which is 14.8 °C and 24.5 °C higher than that irradiated by NIR-II laser at 1.0 W/cm^2^ and 0.5 W/cm^2^, respectively (Fig. 3c). In addition, the photothermal effects of Au@PDA, Au@PDA-PEG and Au@PDA-PEG-Ab were compared. At the same concentration and laser power, Au@PDA solution is about 3 °C higher than PEGylated Au nanoparticles (Au@PDA-PEG) after NIR-II irradiation for 4 min (Fig. 3d, e), suggesting that surface modification with polymers may slightly affect the photothermal performance of Au nanoparticles. However, modification of PEGylated Au nanoparticles with antibody did not have significant impact on the photothermal property of nanoparticles. All three kinds of nanoparticles exhibit excellent photothermal stability (Fig. 3f), which may be attributed to robust PDA coating [37, 38, 49].

### In vitro investigation of cell targeting and cytotoxicity

**Fig. 4:**
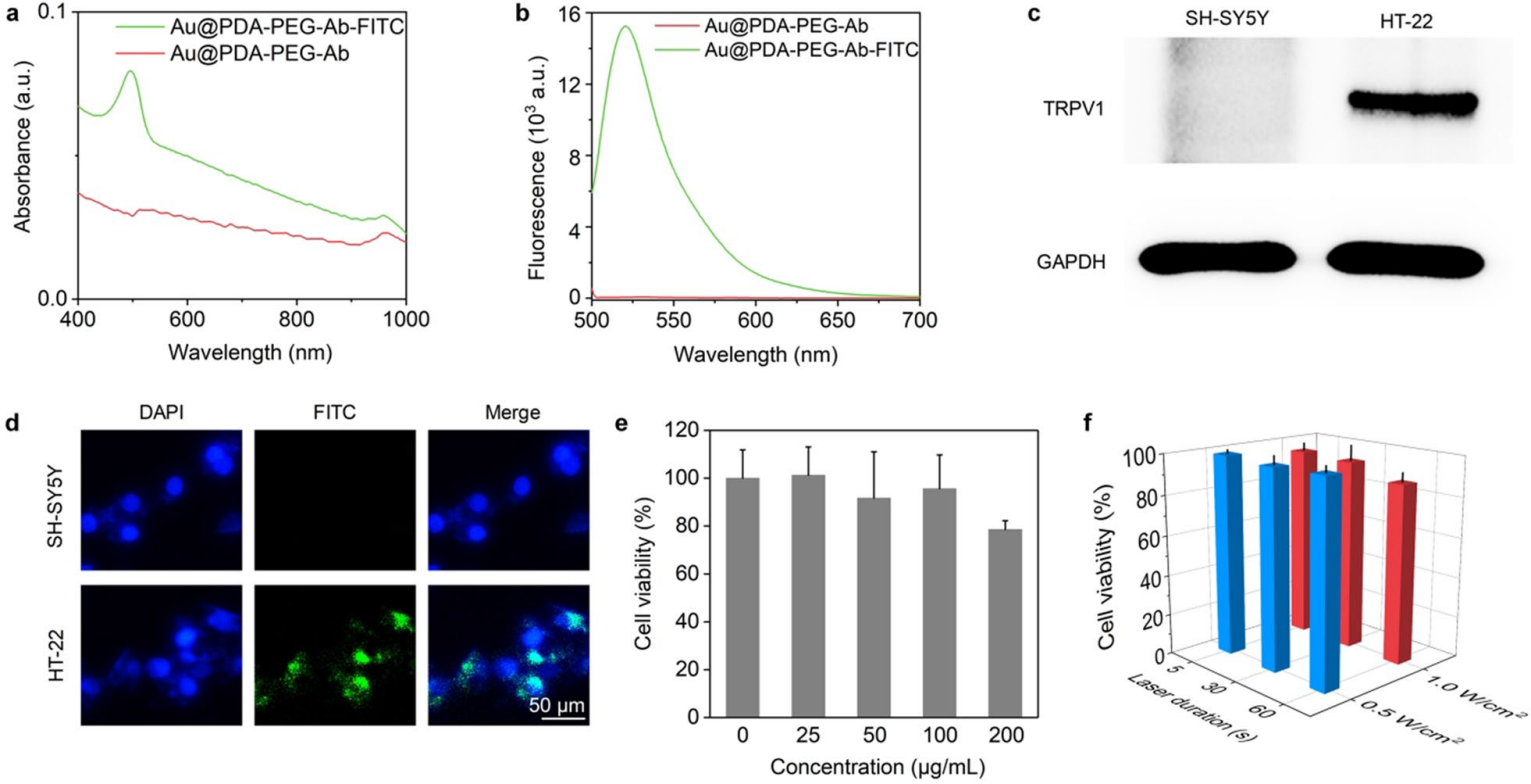
Cellular uptake and cytotoxicity of Au nanoparticles. **a** UV–vis spectra of FITC modified and unmodified Au nanoparticles. **b** Fluorescence spectra of FITC modified and unmodified Au nanoparticles. **c** Western blotting for TRPV1 from SH-SY5Y and HT-22 cells. **d** Cellular uptake of FITC modified Au nanoparticles. **e** Cell viability of HT-22 treated with different concentrations of Au@PDA-PEG-Ab for 24 h. **f** Cell viability of HT-22 treated with NIR-II laser irradiation of different power intensities and laser duration

To examine the ability of the antibody modified-nanoparticles to specifically bind the TRPV1 receptor on the neuronal cell membrane, nanoparticles were labelled with fluorescent probe FITC. The UV–vis spectra revealed that the FITC-labelled nanoparticles have characteristic absorption peak at 495 nm (Fig. 4a). Fluorescence spectroscopy showed that the fluorescent emission peak of FITC-labelled nanoparticles is 520 nm with excitation at 490 nm (Fig. 4b). These results suggest that FITC had been successfully attached to the nanoparticles. ND7/23 cells were most often applied in previous studies to explore the influence of nanoparticle-mediated photothermal stimulation on neuronal activity [28, 34]. However, ND7/23 cells only represent the stimulus–response characteristics of peripheral neurons. The hippocampal neuronal cell line HT-22 and the immortalized human glioblastoma cell line SH-SY5Y cells are two well-established central neurons. The immunoblotting results show that the cellular expression of TRPV1 was abundant in HT-22 but scarce in SH-SY5Y (Fig. 4c), which is consistent with previous reports [6, 50, 51]. Therefore, these two cell lines were chosen as exemplary neurons to examine the effects of photothermal stimulation on central neurons. As shown in Fig. 4d, the fluorescently labelled Au nanoparticles could effectively adsorb to HT-22 cells while no significant fluorescence signal was observed on the surface of SH-SY5Y cells, indicating high targetability of antibody-conjugated Au nanoparticle to TRPV1 on neurons. Then, cytotoxicity experiments were performed to investigate the potential neurotoxicity of Au nanoparticles. Au nanoparticles below 100 μg/mL had no significant toxic effect on HT-22 cells. The survival rate of HT-22 cells was still around 78.5% even the concentration of nanoparticles increased to 200 μg/mL. To evaluate the potentially detrimental effect of laser irradiation during photothermal stimulation, CCK-8 was also used to evaluate the cell viability of HT-22 upon NIR-II laser irradiation. When the concentration of Au nanoparticles was fixed at 50 μg/mL, the cell survival rate remained about 88% even when the irradiation time was extended to 60 s and the irradiation power was increased to 1.0 W/cm^2^ (Fig. 4f). If the laser power or irradiation time was reduced, the cell viability increased further to over 94%. These results demonstrated that Au nanoparticles possess excellent biocompatibility and do not cause significant damage to neurons during brief NIR-II stimulation.

### Photothermal stimulation of neurons in vitro and in vivo

**Fig. 5:**
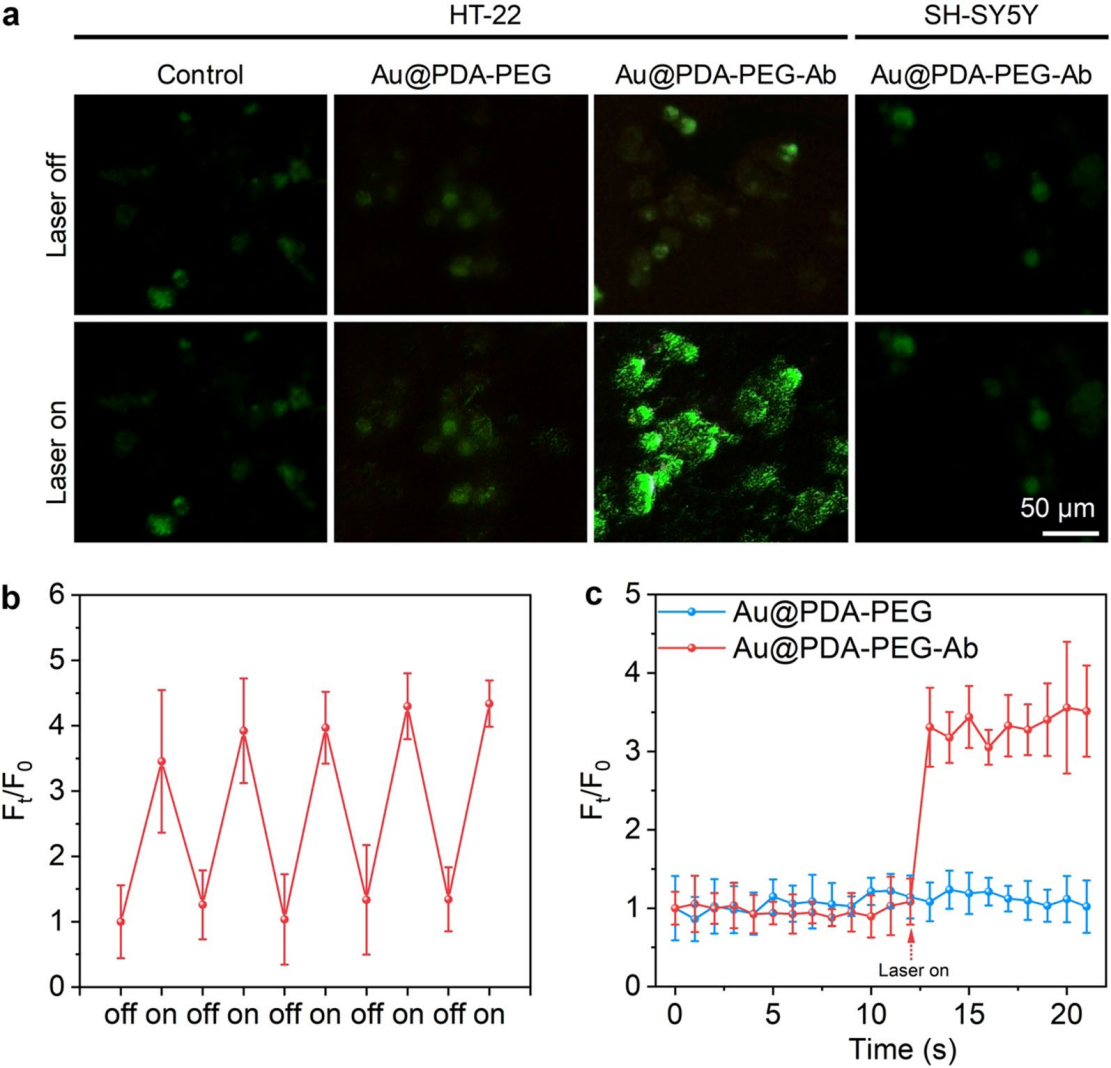
NIR-II photothermal activation of TRPV1 in HT-22 and SH-SY5Y neurons. **a** Fluorescent images of HT-22 or SH-SY5Y cells treated with Au@PDA-PEG or Au@PDA-PEG-Ab before and after laser irradiation at 1064 nm (0.5 W/cm^2^) for 1 s. **b** Changes in the Fluo-4 fluorescence intensity with the 1064 nm laser switching on and off at 1 s intervals. **c** The fluorescence intensity of Fluo-4 as a function of laser irradiation time

To examine the effect of Au nanoparticle-mediated photothermal effects on neuronal activity in vitro, Ca^2+^ indicator (Fluo-4 AM) under NIR-II stimulation. NIR-II irradiation was applied above the culture plates with a distance of less than 0.5 cm. Fluorescence micrographs of HT-22 neurons cultured with Au@PDA-PEG-Ab shows significant Ca^2+^ influx upon NIR-II irradiation for 1 s (Fig. 5a). On the contrast, application of NIR-II irradiation with PBS or Au@PDA-PEG did not induce distinct Ca^2+^ influx. Besides, SH-SY5Y cells also did not show visible Ca^2+^ influx in the presence of Au@PDA-PEG and NIR-II irradiation. The temperature of the Au nanoparticle dispersion under 1-s NIR-II irradiation was far from 43 °C, the threshold to activate TRPV1 ion channel [34, 52]. Therefore, our study confirmed that the activation of TPRV1 was mostly due to localized heat dissipated from the surface of photothermal nanotransducer [34, 53]. Then, the effects of repeated on/off or continuous light stimulation on neuronal activity were investigated. When the near-infrared light source was switched on and off at a frequency of 1 s, the Ca^2+^ movements across cells can be precisely regulated (Fig. 5b and Additional file 1: Fig. S6). After continuous light stimulation, increased intracellular Ca^2+^ signal reach plateau in 1 s and last for 9 s (Fig. 5c and Additional file 1: Fig. S7), comparable to other photothermal transducer [34]. Although the gradual increase in Ca^2+^ signals were observed in real-time, it can’t be directly recorded due to the limited temporal resolution of the fluorescence microscopy used. These data indicated that the Ca^2+^ movements across neuronal membranes can be finely regulated by NIR-II irradiation with the assistance of targeted photothermal nanotransducers.

**Fig. 6:**
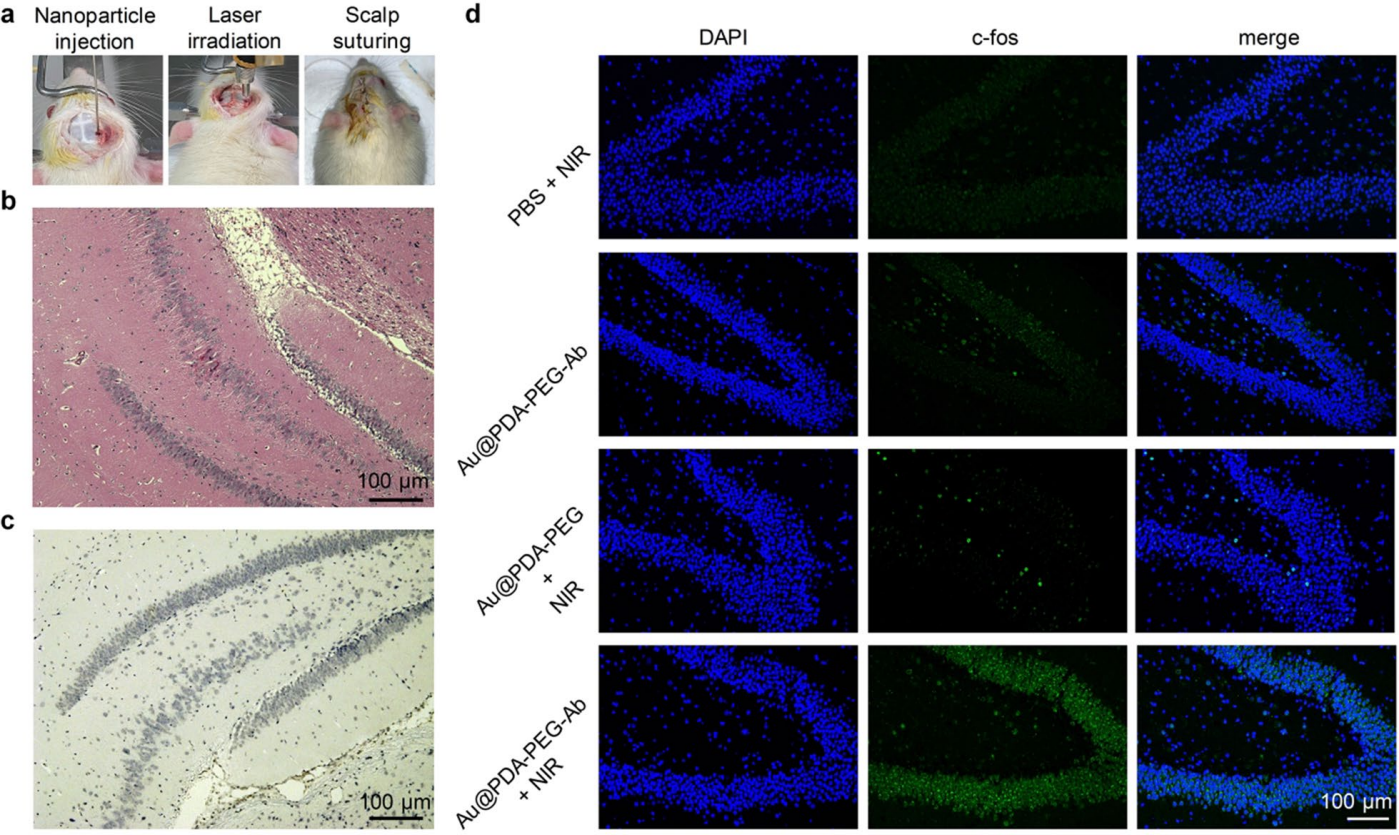
In vivo NIR neural stimulation. **a** Procedures for in vivo neural stimulation. **b** H&E and **c** TUNEL staining of hippocampal slices after treated with Au@PDA-PEG-Ab and NIR-II laser irradiation. **d** C-fos staining of hippocampal slices after Au nanoparticles and NIR-II laser irradiation (1.0 W/cm^2^)

Next, we further investigated the effect of Au nanoparticles-assisted photothermal stimulation of deep brain in vivo. Because of the improved light penetration of NIR-II, we envisaged that it can stimulate deep brain structure. Hippocampus, which located 5 mm beneath the rat cortex was chosen as stimulation site for in vivo experiments (Fig. 6a). Repeated short-pulse NIR-II irradiation were applied to activate neurons without causing significant damage to neurons, as demonstrated by H&E and TUNEL staining (Fig. 6b, c). In accordance with previous results [54], our study confirmed that there was abundant TRPV1 expression in the pyramidal cell layer of hippocampus (Additional file 1: Fig. S7), laying foundation for the photothermal stimulation of hippocampal neurons. C-fos is a kind of protein that reflects the neural activity and its expression is positively correlated with the level of neural activity [41]. Furthermore, it remains a key biomarker to characterize the effects of neurostimulation [55]. Local injection of antibody-modified Au nanoparticles followed by NIR-II irradiation induced local c-fos expression in the targeted deep brain regions but not superficial cortex (Fig. 6d; Additional file 1: Fig. S8). However, neither local injection of unmodified gold nanoparticles nor PBS followed by NIR-II irradiation induced significant c-fos expression. Injection of antibody-modified gold nanoparticles alone also did not induce c-fos expression. Preliminary electrophysiological investigation in brain slice demonstrated that NIR-II irradiation could increase the neuronal action potential firing frequency by approximately 100 percent in the presence of Au@PDA-PEG-Ab (Additional file 1: Fig. S9). In contrast, NIR-II irradiation alone without photothermal transducers did not induce similar effect (Additional file 1: Fig. S10). Overall, our results showed that NIR-II irradiation can stimulate the neural activity of deep brain with the assistance of photothermal transducers.

## Conclusion

In summary, we have demonstrated the reversible NIR-II regulation of neuronal activity using anti-TRPV1 antibody-conjugated Au nanoparticles as biocompatible photothermal nanotransducers. The formed Au@PDA-PEG-Ab showed excellent photothermal conversion efficacy and could specially bind to TRPV1 expressed on hippocampal neurons. In the presence of targeted Au nanoparticles, the TRPV1 receptors of neurons could be activated robustly under NIR-II laser irradiation with a power density as low as 0.5 W/cm^2^. Moreover, we demonstrated the utility of this strategy by stimulating neurons in the 5 mm deep brain of living rat without recruiting neurons of the overlying cortex. Therefore, this platform for non-invasive neuromodulation can be easily extrapolated to other NIR-II photothermal transducers both in vitro and in vivo and serve as a valuable tool in neuroscience and neurology.

## Supplementary Information

**Additional file 1: Fig. S1. **Photographs of Au@PDA solution during preparation. **Fig. S2. **Optimization of PDA-coated Au nanoparticles (Au@PDA). (a) UV-vis spectra of Au nanoparticles prepared with different formulations. Inset: photographs of solutions containing Au nanoparticles with different formulations. (b) Size distributions of different Au nanoparticles. (c) Zeta potentials distributions of different Au nanoparticles. (d) Summary of hydrodynamic sizes and zeta potentials of different Au nanoparticles. **Fig. S3. **TEM characterization of PDA-coated nanoparticles with or without surface PEG modification. **Fig. S4. **Linear time versus -Ln(θ) were obtained from the cooling period in Figure 3a. **Fig. S5. **Fluorescent images of HT-22 cells treated with targeted Au nanoparticles with switching the laser irradiation off and on (1064 nm, 0.5 W/cm^2^) at the interval of 1 s for five cycles. **Fig. S6. **Fluorescent images of HT-22 cells treated with targeted Au nanoparticles with continuous laser irradiation for 9 s (1064 nm, 0.5 W/cm^2^). **Fig. S7. **Expression of TRPV1 in hippocampal slices. **Fig. S8. **C-fos expression in the cortex after Au nanoparticles and 1064 nm laser irradiation (1.0 W/cm^2^). **Fig. S9. **Whole-cell current-clamp recording of action potentials in brain slice in presence of Au@PDA-PEG-Ab before (a&b) and after (c&d) 1064 nm laser irradiation (1.0 W/cm^2^). **Fig. S10. **Whole-cell current-clamp recording of action potentials in brain slice before (a) and after (b) 1064 nm laser irradiation (1.0 W/cm^2^).

## Data Availability

All data are presented in the submitted manuscript.

## Abbreviations

NIR: Near-infrared
PDA: Polydopamine
NIR-II: Near-infrared in second window
TPRV1: Transient receptor potential vanilloid 1
NHS: N-hydroxy succinimide
EDC: 1-Ethyl-3-(3-dimethyl-aminopropyl) carbodiimide
DLS: Dynamic light scattering
CCK-8: Cell counting kit-8
FITC: Fluorescein isothiocyanate isomer I
TEM: Transmission electron microscope
TBST: Tris-buffered saline with Tween
BSA: Bovine serum albumin
