# Supplementary material for: Antibody-conjugated gold nanoparticles as nanotransducers for second near-infrared photo-stimulation of neurons in rats
Source: Nano Converg. 2022 Mar 21;9:13. doi: 10.1186/s40580-022-00304-y (PMC8938552; doi:10.1186/s40580-022-00304-y)
Supplement: Supplementary file 1 — Additional file 1: Fig. S1. Photographs of Au@PDA solution during preparation. Fig. S2. Optimization of PDA-coated Au nanoparticles (Au@PDA). (a) UV-vis spectra of Au nanoparticles prepared with different formulations. Inset: photographs of solutions containing Au nanoparticles with different formulations. (b) Size distributions of different Au nanoparticles. (c) Zeta potentials distributions of different Au nanoparticles. (d) Summary of hydrodynamic sizes and zeta potentials of different Au nanoparticles. Fig. S3. TEM characterization of PDA-coated nanoparticles with or without surface PEG modification. Fig. S4. Linear time versus -Ln(θ) were obtained from the cooling period in Figure 3a. Fig. S5. Fluorescent images of HT-22 cells treated with targeted Au nanoparticles with switching the laser irradiation off and on (1064 nm, 0.5 W/cm2) at the interval of 1 s for five cycles. Fig. S6. Fluorescent images of HT-22 cells treated with targeted Au nanoparticles with continuous laser irradiation for 9 s (1064 nm, 0.5 W/cm2). Fig. S7. Expression of TRPV1 in hippocampal slices. Fig. S8. C-fos expression in the cortex after Au nanoparticles and 1064 nm laser irradiation (1.0 W/cm2). Fig. S9. Whole-cell current-clamp recording of action potentials in brain slice in presence of Au@PDA-PEG-Ab before (a&b) and after (c&d) 1064 nm laser irradiation (1.0 W/cm2). Fig. S10. Whole-cell current-clamp recording of action potentials in brain slice before (a) and after (b) 1064 nm laser irradiation (1.0 W/cm2). [file 40580_2022_304_MOESM1_ESM.docx]

**Additional file 1**

**Antibody-conjugated gold nanoparticles** **as nanotransducers for second near-infrared photo-stimulation of neurons in rats**

Jiansheng Liu^1,2#^, Jiajia Li^3#^, Shu Zhang^1#^, Mengbin Ding^2^, Ningyue Yu^2^, Jingchao Li^2*^, Xiuhui Wang^4*^, Zhaohui Li^1*^

^1^Department of Neurology, Zhuhai People’s Hospital, Zhuhai Hospital of Jinan University, Zhuhai, Guangdong 519000, PR China

^2^Shanghai Engineering Research Center of Nano-Biomaterials and Regenerative Medicine, College of Chemistry, Chemical Engineering and Biotechnology, Donghua University, Shanghai 201620, PR China.

^3^Department of Neurology, Shanghai Eighth People's Hospital, Shanghai 200233, PR China

^4^Institute of Translational Medicine, Shanghai University, Shanghai 200011, PR China

^#^These authors contributed equally to this work.

^*^Corresponding authors: jcli@dhu.edu.cn (J. Li), blackrabbit@shu.edu.cn (X. Wang), Lzh6207@126.com (Z. Li)


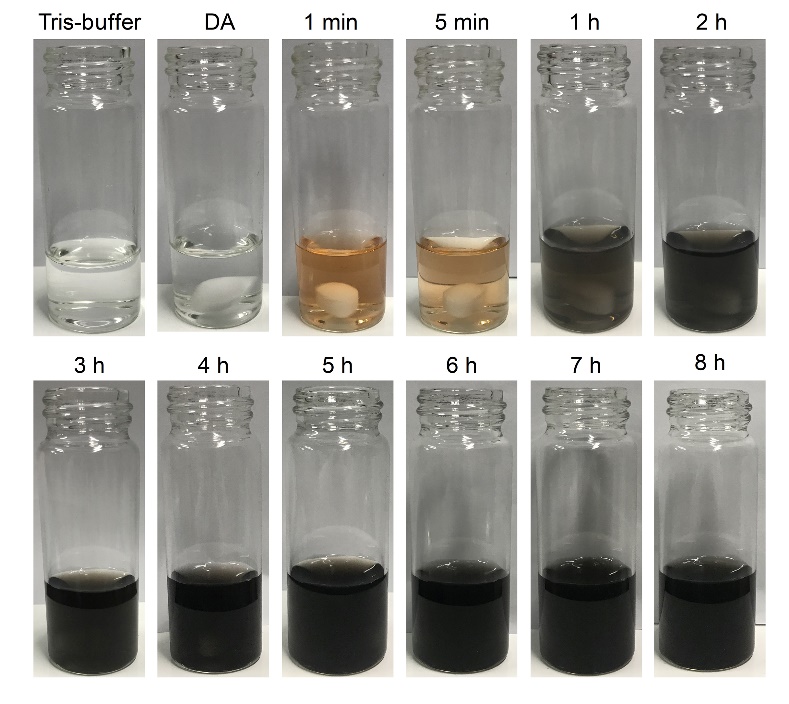


**Fig. S1.** Photographs of Au@PDA solution during preparation.


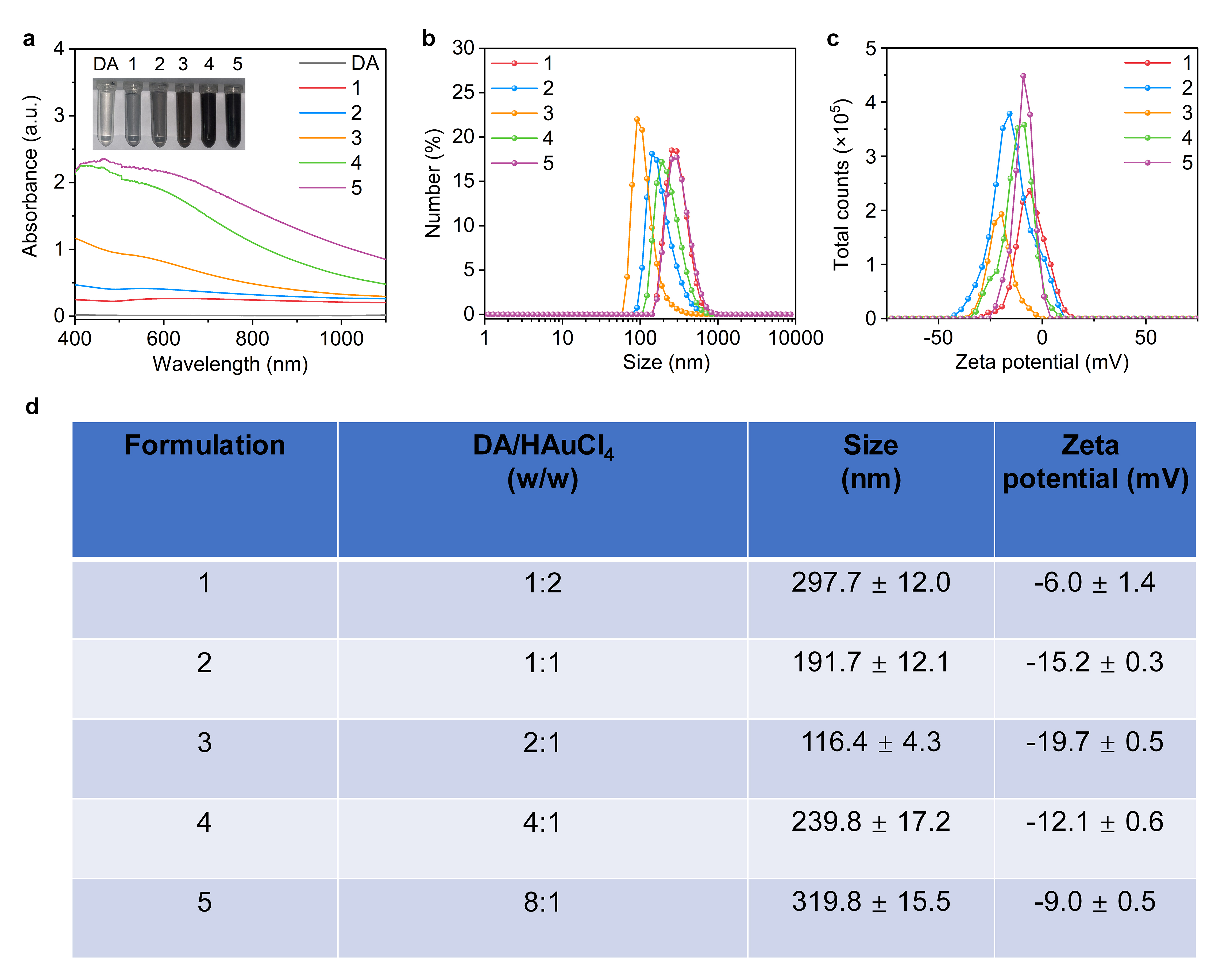


**Fig. S2.** Optimization of PDA-coated Au nanoparticles (Au@PDA). (a) UV-vis spectra of Au nanoparticles prepared with different formulations. Inset: photographs of solutions containing Au nanoparticles with different formulations. (b) Size distributions of different Au nanoparticles. (c) Zeta potentials distributions of different Au nanoparticles. (d) Summary of hydrodynamic sizes and zeta potentials of different Au nanoparticles.


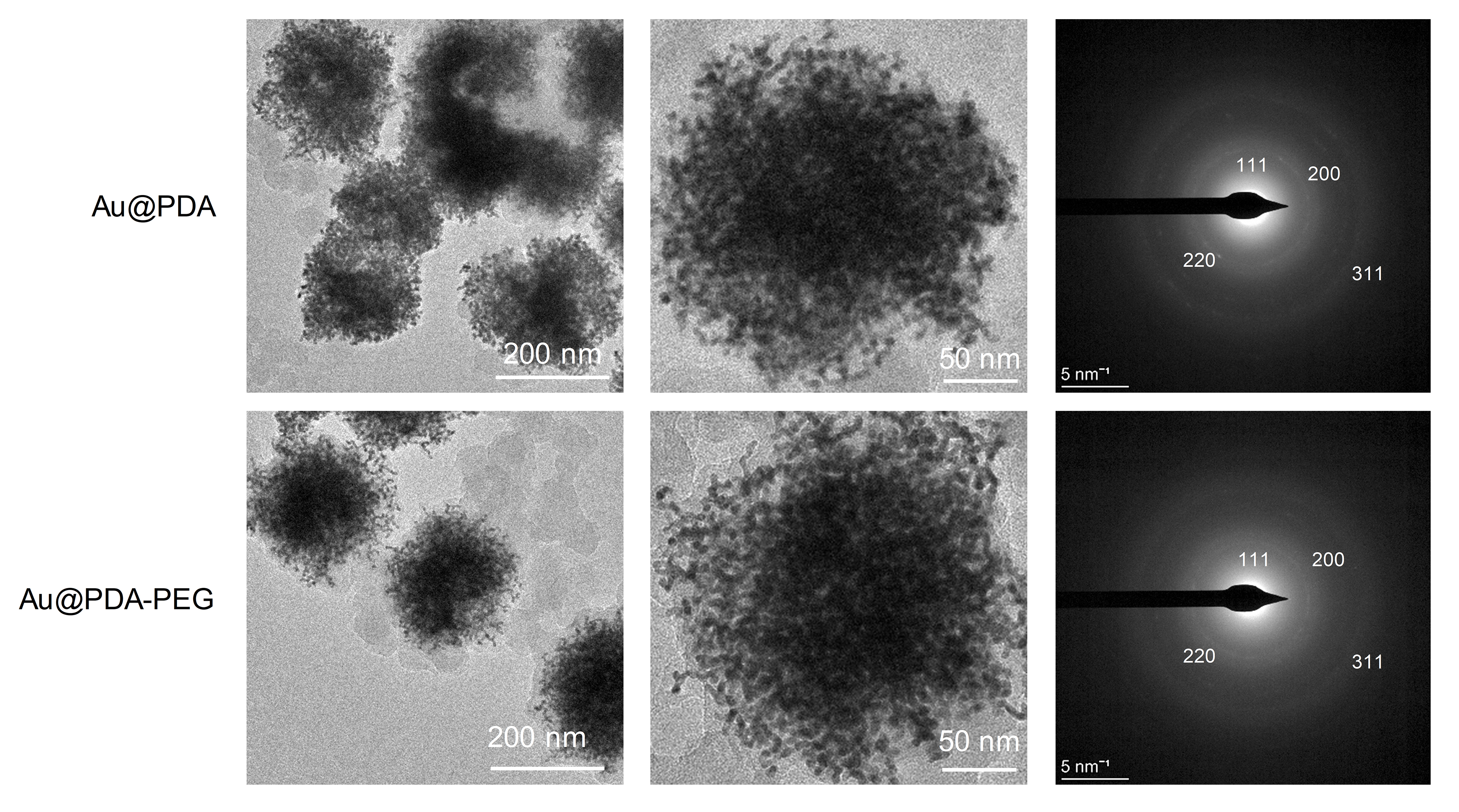


**Fig. S3.** TEM characterization of PDA-coated nanoparticles with or without surface PEG modification.


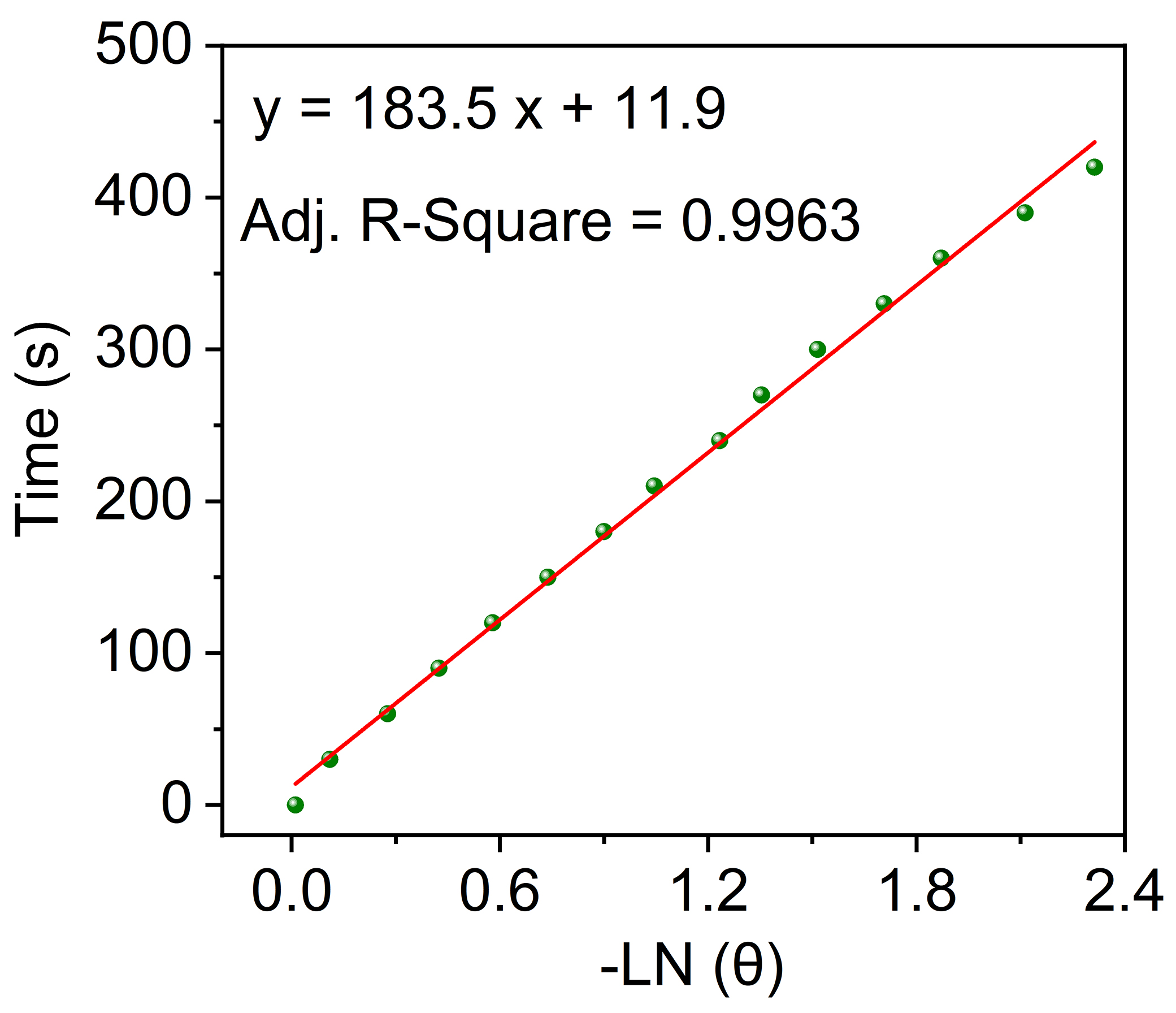


**Fig. S4.** Linear time versus -Ln(θ) were obtained from the cooling period in Figure 3a.

**
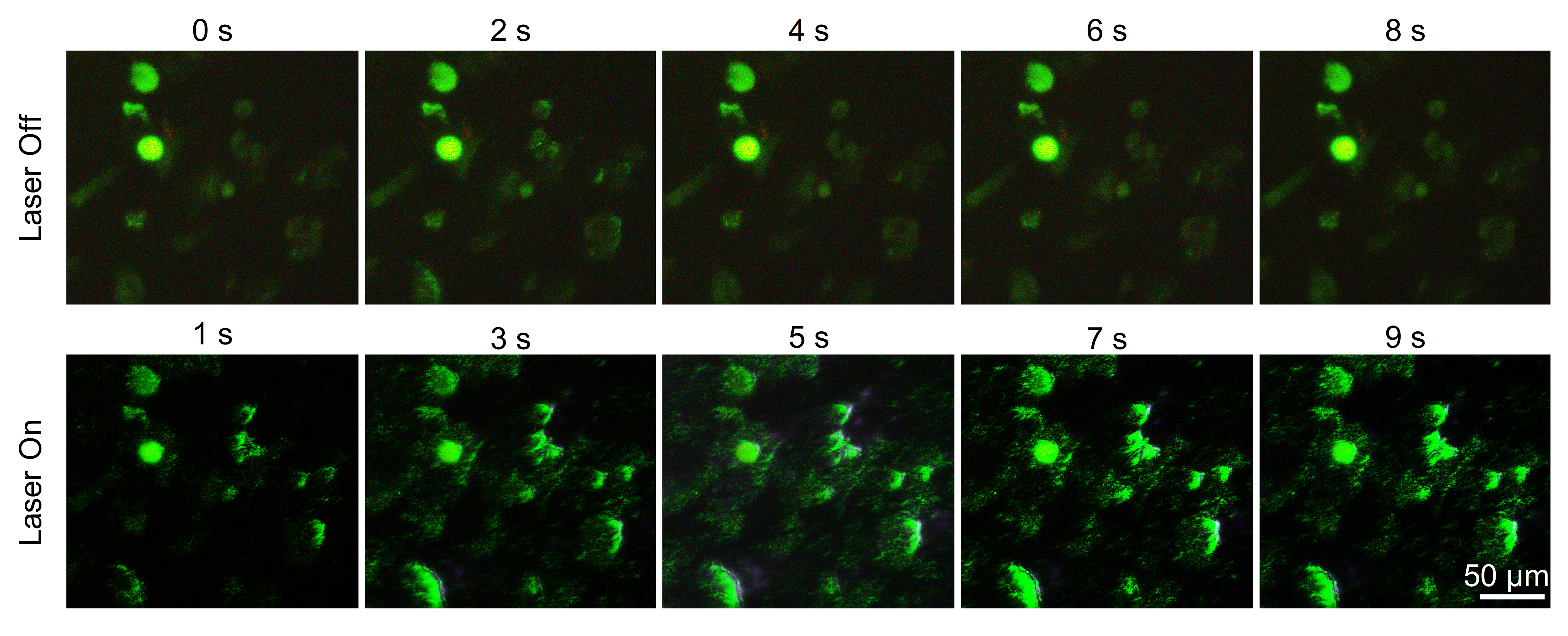
**

**Fig. S5.** Fluorescent images of HT-22 cells treated with targeted Au nanoparticles with switching the laser irradiation off and on (1064 nm, 0.5 W/cm^2^) at the interval of 1 s for five cycles.

**
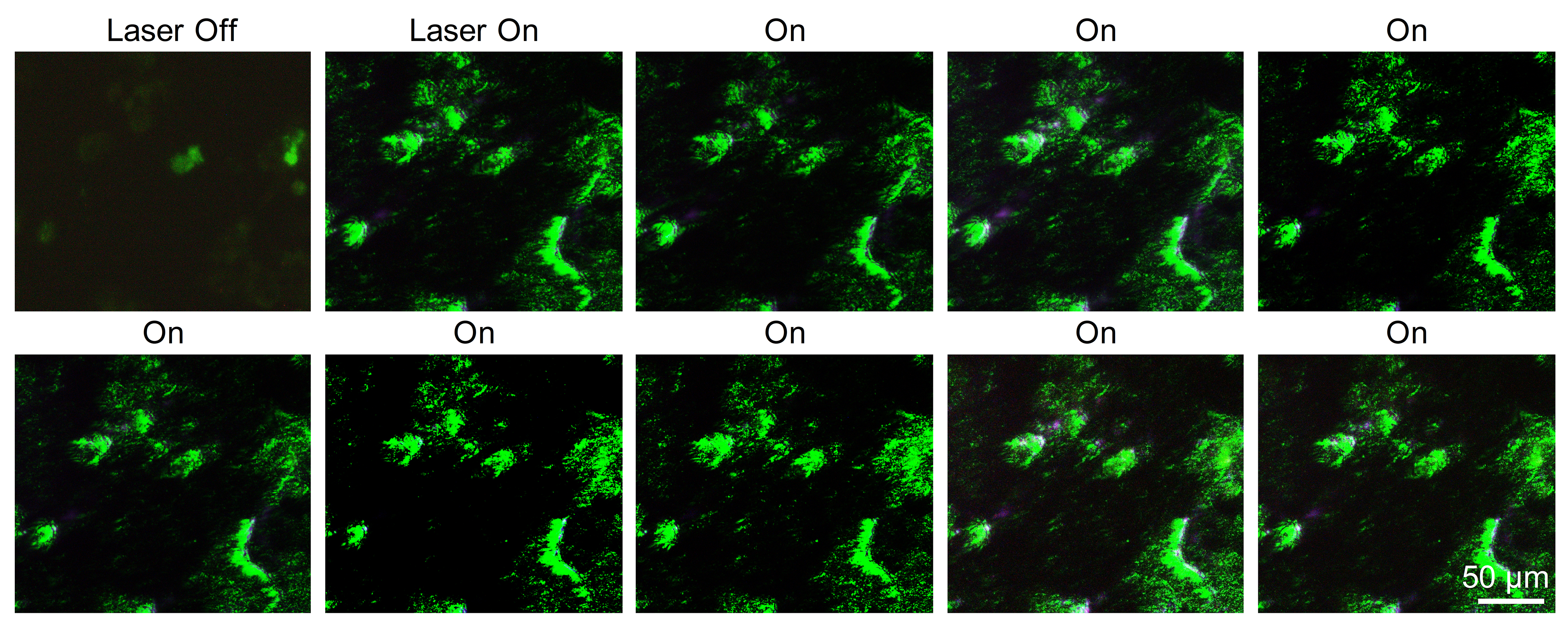
**

**Fig. S6.** Fluorescent images of HT-22 cells treated with targeted Au nanoparticles with continuous laser irradiation for 9 s (1064 nm, 0.5 W/cm^2^).

**
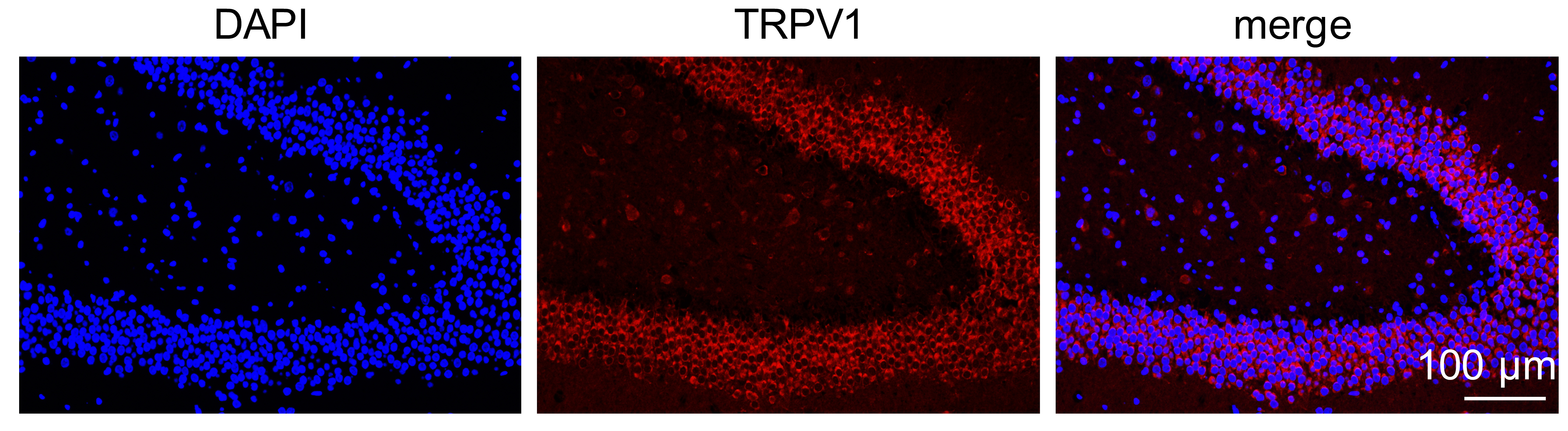
**

**Fig. S7.** Expression of TRPV1 in hippocampal slices.

**
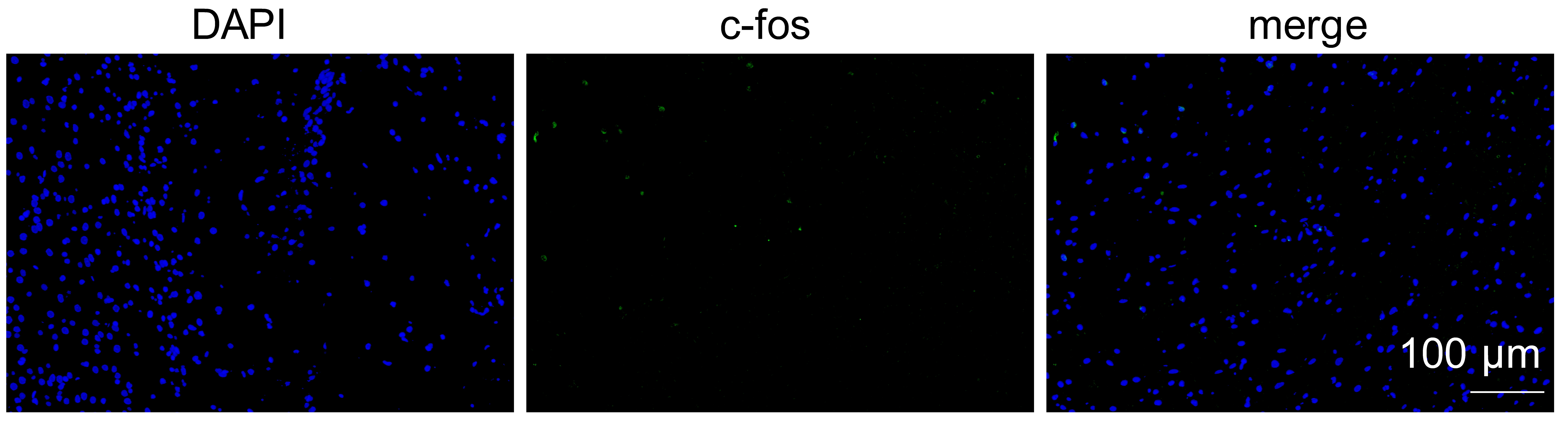
**

**Fig. S8.** C-fos expression in the cortex after Au nanoparticles and 1064 nm laser irradiation (1.0 W/cm^2^).


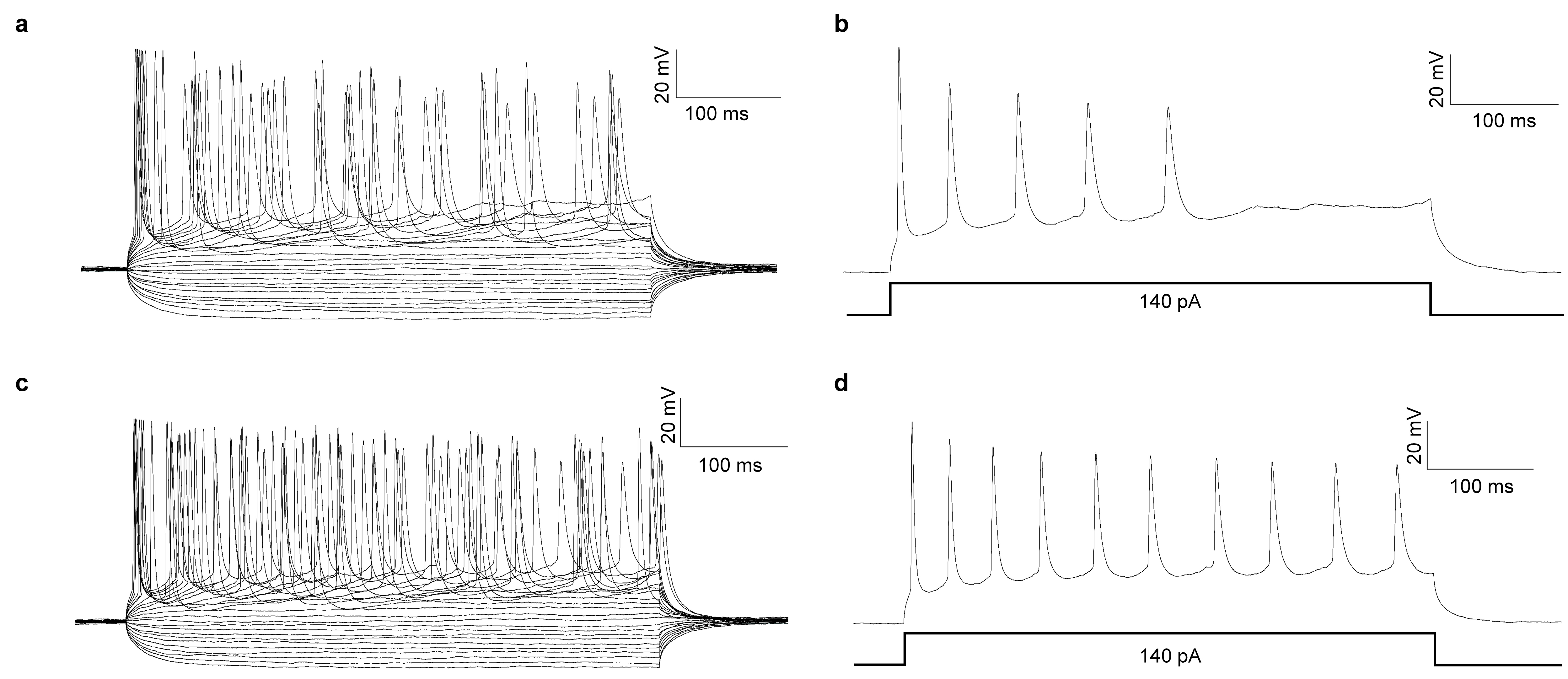


**Fig. S9.** Whole-cell current-clamp recording of action potentials in brain slice in presence of Au@PDA-PEG-Ab before (a&b) and after (c&d) 1064 nm laser irradiation (1.0 W/cm^2^).


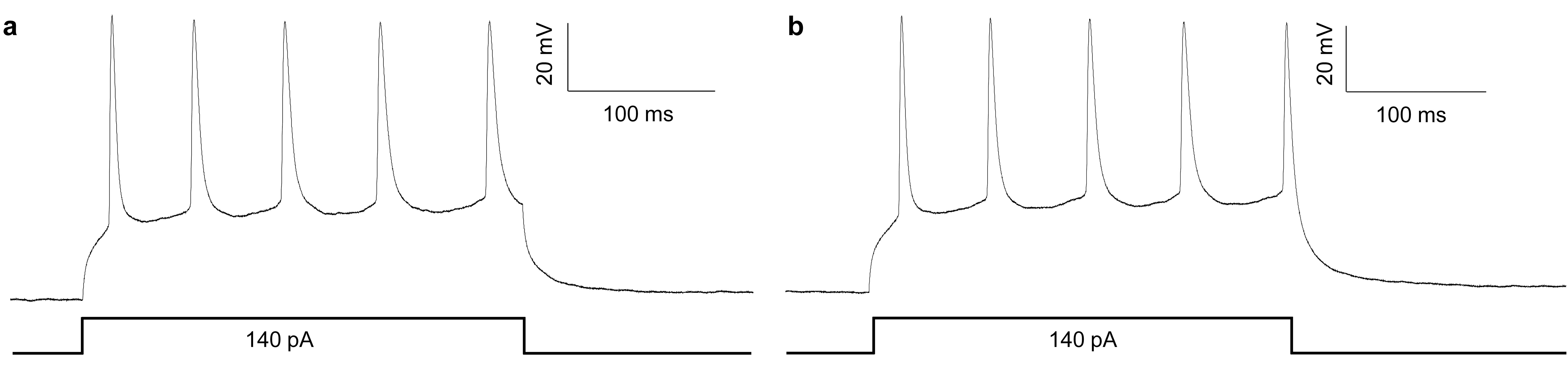


**Fig. S10.** Whole-cell current-clamp recording of action potentials in brain slice before (a) and after (b) 1064 nm laser irradiation (1.0 W/cm^2^).
